# Decreased Brain Levels of Vitamin B12 in Aging, Autism and Schizophrenia

**DOI:** 10.1371/journal.pone.0146797

**Published:** 2016-01-22

**Authors:** Yiting Zhang, Nathaniel W. Hodgson, Malav S. Trivedi, Hamid M. Abdolmaleky, Margot Fournier, Michel Cuenod, Kim Quang Do, Richard C. Deth

**Affiliations:** 1 Department of Pharmaceutical Sciences, Northeastern University, Boston, MA, 02115, United States of America; 2 Department of Surgery, Laboratory of Nutrition and Metabolism at BIDMC, Harvard Medical School, Boston, MA, 02215, United States of America; 3 Department of Pharmaceutical Sciences, Nova Southeastern University College of Pharmacy, Fort Lauderdale, FL, 33328, United States of America; 4 Department of Medicine (Biomedical Genetics Section), Genetics & Genomics, Boston University School of Medicine, Boston, MA, 02118, United States of America; 5 Center for Psychiatric Neuroscience, Department of Psychiatry, Lausanne University Hospital, Lausanne, Switzerland; Bauer Research Foundation, UNITED STATES

## Abstract

Many studies indicate a crucial role for the vitamin B_**12**_ and folate-dependent enzyme methionine synthase (MS) in brain development and function, but vitamin B_12_ status in the brain across the lifespan has not been previously investigated. Vitamin B_12_ (cobalamin, Cbl) exists in multiple forms, including methylcobalamin (MeCbl) and adenosylcobalamin (AdoCbl), serving as cofactors for MS and methylmalonylCoA mutase, respectively. We measured levels of five Cbl species in postmortem human frontal cortex of 43 control subjects, from 19 weeks of fetal development through 80 years of age, and 12 autistic and 9 schizophrenic subjects. Total Cbl was significantly lower in older control subjects (> 60 yrs of age), primarily reflecting a >10-fold age-dependent decline in the level of MeCbl. Levels of inactive cyanocobalamin (CNCbl) were remarkably higher in fetal brain samples. In both autistic and schizophrenic subjects MeCbl and AdoCbl levels were more than 3-fold lower than age-matched controls. In autistic subjects lower MeCbl was associated with decreased MS activity and elevated levels of its substrate homocysteine (HCY). Low levels of the antioxidant glutathione (GSH) have been linked to both autism and schizophrenia, and both total Cbl and MeCbl levels were decreased in glutamate-cysteine ligase modulatory subunit knockout (GCLM-KO) mice, which exhibit low GSH levels. Thus our findings reveal a previously unrecognized decrease in brain vitamin B_12_ status across the lifespan that may reflect an adaptation to increasing antioxidant demand, while accelerated deficits due to GSH deficiency may contribute to neurodevelopmental and neuropsychiatric disorders.

## Introduction

**Fig 1:**
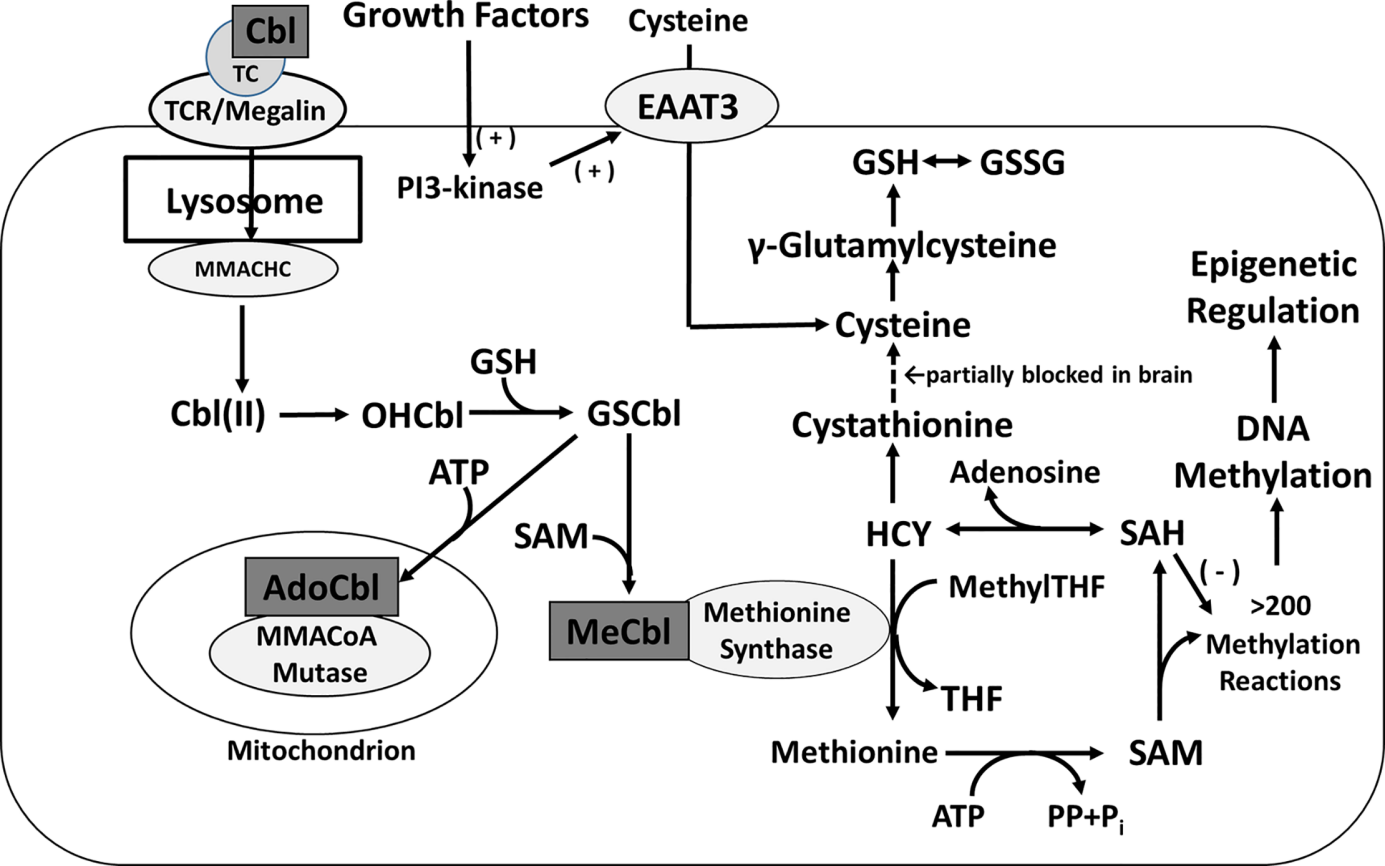
Cobalamin-related redox metabolic pathways in neuronal cells. Endocytosis brings TC-bound Cbl species to lysosomes where axial ligands are removed by MMACHC and MeCbl or AdoCbl are subsequently formed by SAM and ATP-dependent pathways, respectively. MeCbl is a required cofactor for methionine synthase, whose activity supports a large number of methylation reactions, including DNA methylation, as well as dopamine-stimulated phospholipid methylation, carried out by the D4 dopamine receptor (D4R). AdoCbl supports MMACoA mutase in mitochondria. Cysteine, which is rate-limiting for GSH synthesis, can be provided either by cellular uptake via the cysteine/glutamate transporter EAAT3 (excitatory amino acid transporter 3) or by transsulfuration of HCY via cystathionine. The latter pathway is restricted in human brain, increasing the importance of growth factor-dependent cysteine uptake by EAAT3.

Metabolically active forms of vitamin B_12_, methylcobalamin (MeCbl) and adenosylcobalamin (AdoCbl), serve as essential cofactors for two reactions: MeCbl for folate-dependent methylation of HCY to methionine by methionine synthase (MS) in the cytoplasm, and AdoCbl for conversion of methylmalonylCoA to succinylCoA by methylmalonyl CoA mutase in mitochondria (Fig 1) [1,2]. Since MS activity determines the ratio of the methyl donor S-adenosylmethionine (SAM) to the endogenous methylation inhibitor S-adenosylhomocysteine (SAH), MeCbl is poised to influence hundreds of SAM-dependent methylation reactions, affecting nearly every aspect of metabolism. Important among these reactions is methylation of DNA and histones, which combine to exert dynamic epigenetic control over gene expression [3]. MeCbl is also required for dopamine-stimulated phospholipid methylation, a unique activity of D4 dopamine receptors [4], which depends upon MS activity [5] and has been proposed to play an important role in neuronal synchronization and attention [6]. Genetic variants of the D4 receptor have been linked to attention-deficit hyperactivity disorder (ADHD) [7,8], schizophrenia risk [9,10], and drug addiction [9], as well as to human longevity [11].

Vitamin B_12_ is only synthesized by certain bacteria and humans obtain it from animal source foods such as meat, dairy, eggs, and fish.[12]. A series of chaperones, transport proteins and their receptors (e.g. haptocorrin, intrinsic factor, cubilin, amnionless and megalin) protect vitamin B_12_ and facilitate its GI absorption and renal reabsorption for its retention. In the general circulation vitamin B_12_ primarily exists bound to transcobalamin (TC) [13]. As illustrated in Fig 1, cell surface receptors (TC receptor and/or megalin) bring the Cbl·TC complex into lysosomes where Cbl is processed by MMACHC (methylmalonic aciduria type C and homocystinuria, also known as CblC). MMACHC carries out dealkylation of alkylCbls and decyanation of cyanocobalamin (CNCbl) in glutathione (GSH)-dependent and NADPH-dependent reactions, respectively [2]. Formation of active cofactors MeCbl and AdoCbl is then carried out by MMACHC in conjunction with MMADHC (methylmalonic aciduria type D and homocystinuria, also known as CblD) in the cytoplasm and mitochondria, respectively.

The brain exists within a distinct compartment and levels of metabolic resources, including vitamin B_12_, are reflective of their transport into and out of cerebral spinal fluid (CSF) across the neuroepithelial barrier in the choroid plexus. While factors responsible for vitamin B_12_ entry into brain have not been fully elucidated, cubilin and megalin, which combine to participate in transport of vitamin B_12_ in other tissues, are expressed in the choroid plexus [14,15], and a role for amnionless has been postulated based upon disturbed vitamin B_12_ transport into the brain in a patient with a mutation causing Imerslund-Gräsbeck syndrome [16]. While diet or genetic defects in transport/ processing can affect systemic vitamin B_12_ availability [17,18], there have been relatively few direct studies of vitamin B_12_ status in human brain [19,20] and none have provided a comprehensive analysis of different Cbl species.

Methylation of DNA and histone proteins complexly regulates gene expression and this form of epigenetic regulation is particularly important during development, including pre- and postnatal brain development [21]. Neural tube defects, as well as Rett and Angelman/Prader-Willi neurodevelopmental syndromes are linked to defects in methylation-dependent epigenetic regulation [22–24]. Turnover of DNA methylation marks is very fast in prefrontal cortex during fetal development but is 2–3 orders lower during childhood and later life [25]. We previously showed that the level of MS mRNA in human prefrontal cortex decreases several hundred-fold across the lifespan, indicating a dynamic role for vitamin B_12_-dependent MS activity in brain development and function, and MS mRNA levels were prematurely decreased in autistic subjects [26]. Abnormal DNA methylation [27, 28] has been reported in postmortem brain of autistic subjects, in conjunction with low levels of the antioxidant GSH and elevated markers of oxidative stress [29,30]. Increased oxidative stress and impaired methylation have also been implicated in schizophrenia [31,32].

We utilized a novel HPLC/electrochemical detection-based assay to quantify individual Cbl species in postmortem human cerebral cortex of control subjects from fetal to 80 yrs of age, as well as autistic and schizophrenic subjects. Changes in Cbl species were compared with the status of methylation and antioxidant pathway metabolites and the influence of decreased GSH production on brain Cbl levels was evaluated in glutamate-cysteine ligase modulatory subunit knockout (GCLM-KO) mice in which GSH synthesis was impaired, leading to a brain GSH level decrease of 60–70% [33]. Our results reveal an unexpected decrease in cortical Cbl and MeCbl levels across the lifespan, as well as premature decreases in both autism and schizophrenia, which were replicated in GCLM-KO mice.

## Materials and Methods

### Tissue sample acquisition

Institutional approval for the use of postmortem brain samples was provided by the Northeastern University IRB (# 04-11-09). Postmortem samples of frontal cerebral cortex (Brodmann areas 9, 10, 44 or 45) were obtained from the Autism Tissue Program, now part of the Autism Brain Network (http://www.autismbrainnet.com), the Australian Brain Bank Network (http://www.austbrainbank.org.au) and the Harvard Brain Tissue Resource Center (http://www.brainbank.mclean.org). Samples included 43 control subjects of different age, from 19 weeks of gestation through 80 yrs (Table A in S1 File), as well as 12 autistic subjects (4–9 yrs) (Table B in S1 File) and 9 schizophrenic subjects (36–49 yrs) (Table C in S1 File). Placenta samples were commercially obtained from Advanced Tissue Services (Phoenix, AZ). All tissues samples were maintained in liquid nitrogen until their use and experiments were completed within four months of their receipt.

### Vitamin B_12_ analysis

Cbl extraction and HPLC mobile phase selection were based on a previously published method [34]. Extraction was performed under dim-red light due to Cbl light sensitivity. Brain tissues were thawed on ice and a 10% homogenate was prepared. 150 μL of ice-cold absolute ethanol was added to 100 μL of each sample homogenate and incubated for 10 min. Protein precipitates were removed by centrifugation at 10,600 RPM for 3 min at 20°C. The resulting supernatant was evaporated to dryness, re-suspended with 300 μL PBS and passed through a syringe-driven filter (0.22 μm). The Cbl extract was then transferred to a conical micro autosampler vial, blown with nitrogen, capped and kept at 4°C in the autosampler cooling tray, covered by aluminum foil to avoid Cbl degradation. 30 μL of sample was injected into an Agilent Eclipse XDB-C8 (3 x 150mm; 3.5 μm) and Agilent Eclipse XDB-C8 (4.6 x 12.5mm; 5 μm) guard column by the autosampler. Samples were eluted using the following step gradient: 0–2 min 0% B, 2–14 min 17% B, 14–19 min 30% B, 24–31 min 58% B, 31–32 min 100% B, then equilibrate column with 0% B for 2 min at a flow rate of 0.6 mL/min. Mobile phase A contained 0.1% acetic acid/acetate buffer titrated to pH 3.5 with NH_4_OH. Mobile phase B was acetonitrile containing 0.1% acetic acid. Cbls were measured using electrochemical detection with an ESA CoulArray with BDD analytical cell model 5040 electrochemical detector at an operating potential of 1000 mV. Examples of chromatograms for cobalamin standards and brain samples are provided (Figures A-E in S2 File). Peak area analysis, based on standard curves generated for each compound, was performed using CoulArray software (version 3.06 ESA analysis program package). Sample Cbl levels were normalized against protein content. Based upon spiked tissue samples, the extraction procedure resulted in recovery of 94.7 +/- 1.8% of tissue Cbl, and replication studies yielded a coefficient of variation of 6.3%.

### Thiol metabolite analysis

Thiol and thioether metabolites were measured using HPLC with electrochemical detection. Brain samples were thawed on ice, and a 10% homogenate was prepared. 50 mL of a 0.4 N perchloric acid solution was added to 200 μL of the sample, and samples were gently blown with nitrogen gas before being centrifuged at 13,000 RPM for 60 min. 100 μL of sample was added to a microautosampler vial, blown with nitrogen gas, capped and loaded at 4°C in the autosampler cooling tray. 10 μL of sample was injected into the HPLC system and measured by electrochemical detection. HPLC columns and running conditions were as same as previously published [35].

### Methionine synthase assay

A 5% homogenate of postmortem brain samples was prepared in lysis buffer at 4°C. The assay was conducted under anaerobic and dark conditions, as previously described [36]. 385 μl of a 5% brain homogenate was mixed with 1 M K_2_HPO_4_, 10 mM HCY, 100 mM DTT, 3.8 mM SAM, adding either 10 μl water or 10 μl of 5 mM OHCbl, in a final volume of 500μl. The assay was initiated by addition of [^14^C-*methyl*] methyltetrahydrofolate, incubated for 60 min at 37°C and terminated by heating at 98°C for 2 min. MS activity was determined by measuring incorporation of ^14^C into methionine, which was separated by passing through a Dowex 1-X8 column.

### GCLM knockout mice studies

GCLM-KO mice were generated from C57Bl/6J mice [37] and kindly provided by TP Dalton (Cincinnati University, Ohio). Experiments were performed in accordance with the guidelines of the Veterinary Office of the Canton de Vaud, Switzerland and approved by the Swiss Federal Food Safety and Veterinary Office (FSVO). Animals were maintained in a temperature-and humidity-controlled environment under a 12-h light–dark cycle with free access to food and water. Animal welfare was checked 3-times per week; mice displaying signs of dysfunction, wounds or important loss of weight were sacrificed. Heterozygous mice were bred and after genotyping, male littermates were decapitated at 40 and 90 days of age and sections of frontal cortex were dissected and frozen at—80°C until analysis of thiol and Cbl levels.

### Statistical analyses

Statistical analyses were carried out using Graph Pad Prism^®^ version 5.01. Results were expressed as mean ± SEM. A two-tailed Student’s t-test and one-way analysis of variance (ANOVA) with Tukey’s *post hoc* test were used to determine statistical significance, using p < 0.05 as a criterion. Correlations were evaluated by Pearson’s correlation coefficient.

## Results

### Frontal cortex Cbl across the lifespan

**Fig 2:**
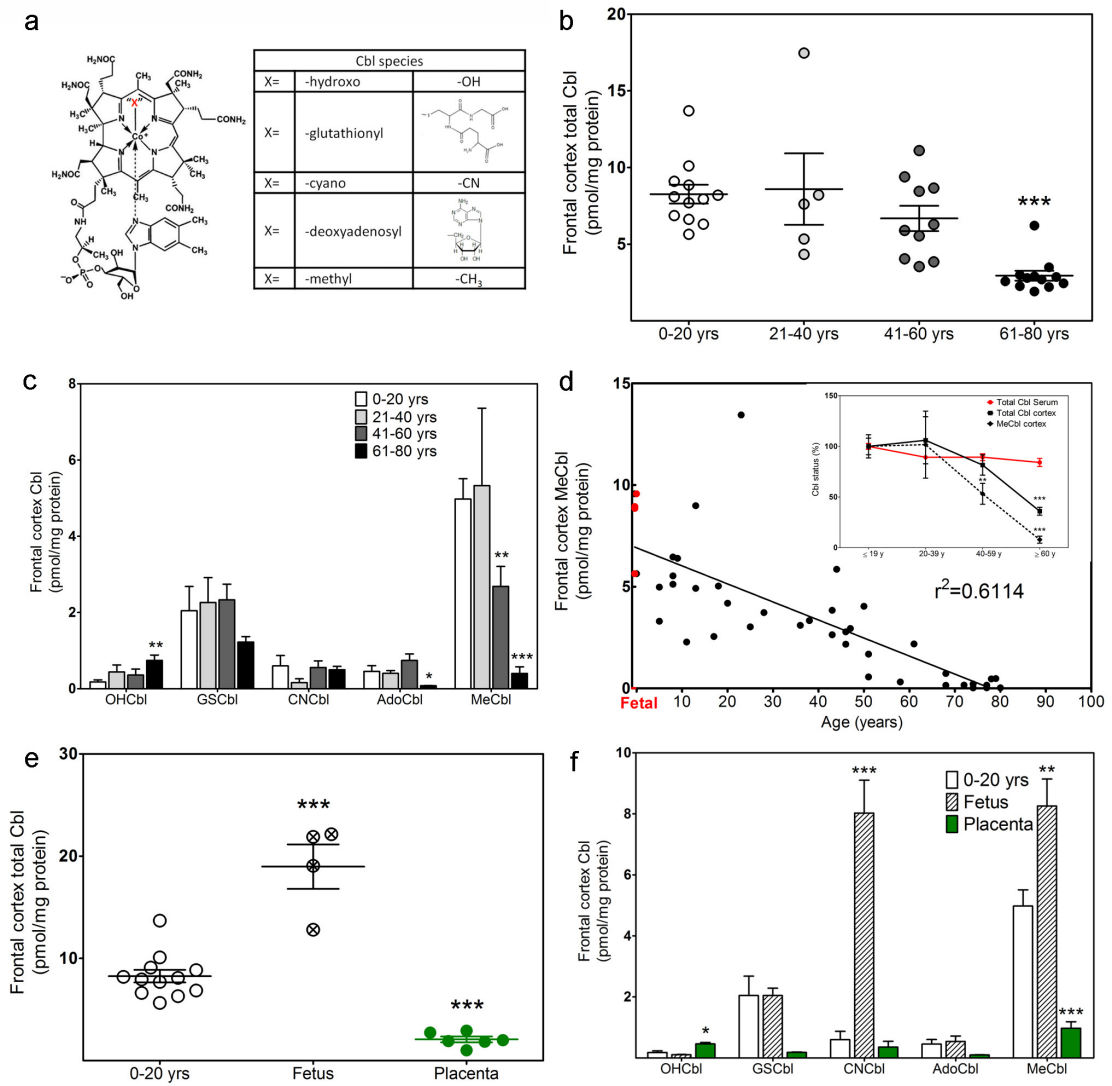
Cobalamin status in human frontal cortex. (a) The general structure of Cbl species in which “X” represents various ligands linked to the cobalt atom, giving rise to the five different Cbl species measured in postmortem frontal cortex. (b) Total Cbl levels in frontal cortex of control subjects divided into four age groups: 0–20 yrs (n = 12), 21–40 yrs (n = 5), 41–60 yrs (n = 10) and 61–80 yrs (n = 12). (c) Levels of five individual Cbl species of control subjects in four age groups. (d) Age-dependent decrease of MeCbl in human frontal cortex (n = 43). Inset: Age trends of serum Cbl, frontal cortex total Cbl and MeCbl. Serum Cbl data is from Ref. 30. (e) Total Cbl levels in placenta (n = 6), frontal cortex of fetal (n = 4) and control (0–20 yrs) subjects (n = 12). (f) Levels of five individual Cbl species in placenta (n = 6), frontal cortex of fetal (n = 4) and control (0–20 yrs) subjects (n = 12). * Indicates a significant difference from 0–20 yrs group (* p < 0.05, ** p < 0.01, *** p < 0.001).

Different species of vitamin B_12_ are distinguished by the ligand attached to the upper face of the corrin ring cobalt atom, and they include MeCbl, AdoCbl, CNCbl, hydroxocobalamin (OHCbl) and glutathionylcobalamin (GSCbl) (Fig 2A). These five Cbl species were detected in postmortem frontal cortex brain samples from subjects grouped by age and we observed that the combined Cbl total was 2.7-fold lower in 61–80 yr old *vs*. 0–20 yr old subjects (Fig 2B). Levels of individual Cbl species were similar in 0–20 and 21–40 yr old control subjects, but significant differences were observed in 41–60 and 61–80 yr old subjects (Fig 2C). Among individual Cbl species, the greatest difference was an age-dependent decline in MeCbl, such that its level in 61–80 yr old subjects was 12.4-fold lower than in 0–20 yr-olds and 6.7-fold lower than 41–60 yr-olds. At younger ages MeCbl was the predominant Cbl species, but its level decreased across the lifespan and was eclipsed by OHCbl, GSCbl and CNCbl in older subjects (61–80 yrs). This progressive age-dependent decrease in frontal cortex MeCbl levels was quasi-linear (r^2^ = 0.61) and MeCbl was negatively correlated with age across the lifespan (p < 0.0001) (Fig 2D). AdoCbl was significantly lower in 61–80 yr old *vs*. 0–20 yr old subjects (p < 0.001), but its level was not correlated with age across the lifespan. In contrast, the level of OHCbl was increased in 61–80 yr old subjects and was positively correlated with age (p = 0.01). This substantial age-dependent decrease in total vitamin B_12_, MeCbl and AdoCbl in frontal cortex contrasts with the comparatively stable level of serum vitamin B_12_ levels, as reported for samples derived from the National Health and Nutrition Examination Survey (NHANES) [38] (Fig 2D, inset). Thus frontal cortex levels of vitamin B_12_ appear to be more dynamically regulated across the lifespan than blood levels.

To investigate possible differences between prenatal and postnatal vitamin B_12_ status, we compared Cbl levels in fetal frontal cortex with levels in young subjects (0–20 yrs). Strikingly, the level of CNCbl was almost 15-fold higher in fetal samples *vs*. young subjects, while the level of MeCbl was 65% higher (Fig 2E). We further examined placental vitamin B_12_ status as a potential source of higher CNCbl for fetal brain. However, total Cbl levels in placenta were 10-fold lower than fetal brain and CNCbl levels were 20-fold lower, making it an unlikely source (Fig 2F).

### Frontal cortex Cbl in autism and schizophrenia

**Fig 3:**
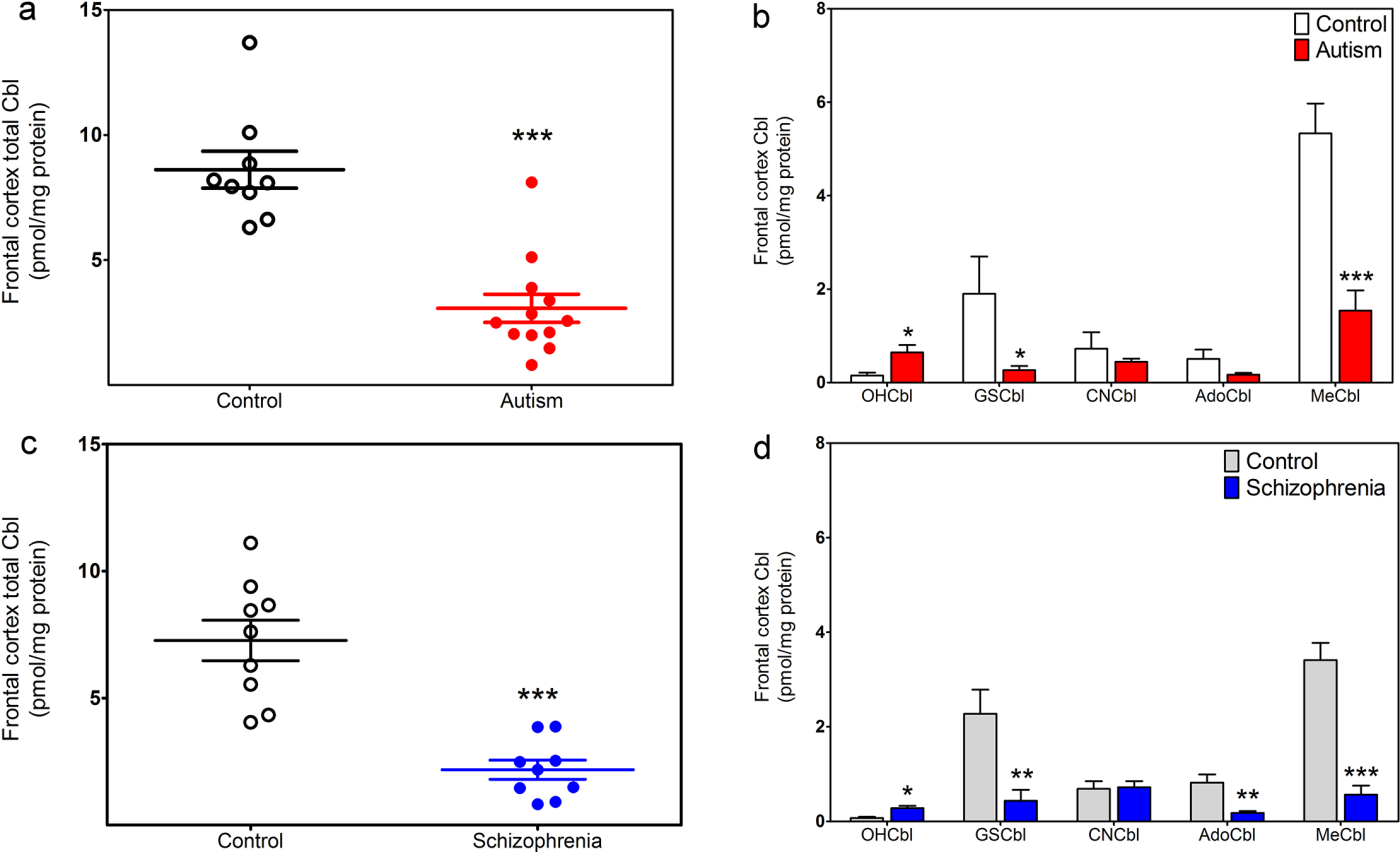
Cobalamin status in autism and schizophrenia. (a) Total Cbl levels in frontal cortex of autistic subjects (n = 12) and aged-matched controls (n = 9). (b) Levels of five individual Cbl species in frontal cortex of autistic subjects (n = 12) and aged-matched controls (n = 9). (c) Total Cbl levels in frontal cortex of schizophrenic subjects (n = 9) and aged-matched controls (n = 9). (d) Levels of five individual Cbl species in frontal cortex of schizophrenic subjects (n = 9) and aged-matched controls (n = 9). * Indicates a significant difference from control group (* p < 0.05, ** p < 0.01, *** p < 0.001).

Cbl levels were analyzed in frontal cortex from young autistic subjects (<10 yrs) and compared to levels in young control subjects (<13 yrs). As illustrated in Fig 3A, the average total Cbl level was 3.1-fold lower in autistic subjects *vs*. controls (3.1 *vs*. 8.9 pmol/mg protein). MeCbl and AdoCbl were each more than 3-fold lower in autistic subjects *vs*. control levels, although the decrease in AdoCbl did not reach statistical significance (p = 0.07) (Fig 3B). Thus the level of frontal cortex Cbl in autistic subjects corresponds to the level of control subjects >50 yrs. Notably, the level of GSCbl was 6-fold lower in autistic subjects, while the level of OHCbl was >3-fold higher, consistent with impairment of GSH-dependent synthesis of MeCbl and AdoCbl.

Cortical Cbl levels were also measured in schizophrenic subjects (ages 36–49 yrs) and compared to levels in control subjects (ages 36–50 yrs). As illustrated in Fig 3C, the average total Cbl level was 3.3-fold lower in schizophrenic subjects (7.3 *vs*. 2.2 pmol/mg protein). MeCbl and AdoCbl were >5-fold lower in schizophrenic subjects *vs*. control levels (Fig 3D). GSCbl was 6-fold lower in schizophrenic subjects, while the level of OHCbl was 3.5-fold higher. Thus both autistic and schizophrenic subjects show similar abnormal patterns of frontal cortex Cbl, including lower levels of both MeCbl and GSCbl. Moreover, the lower levels of total Cbl, MeCbl and AdoCbl are similar to the pattern observed in elderly subjects.

### Methylation and redox metabolites

**Fig 4:**
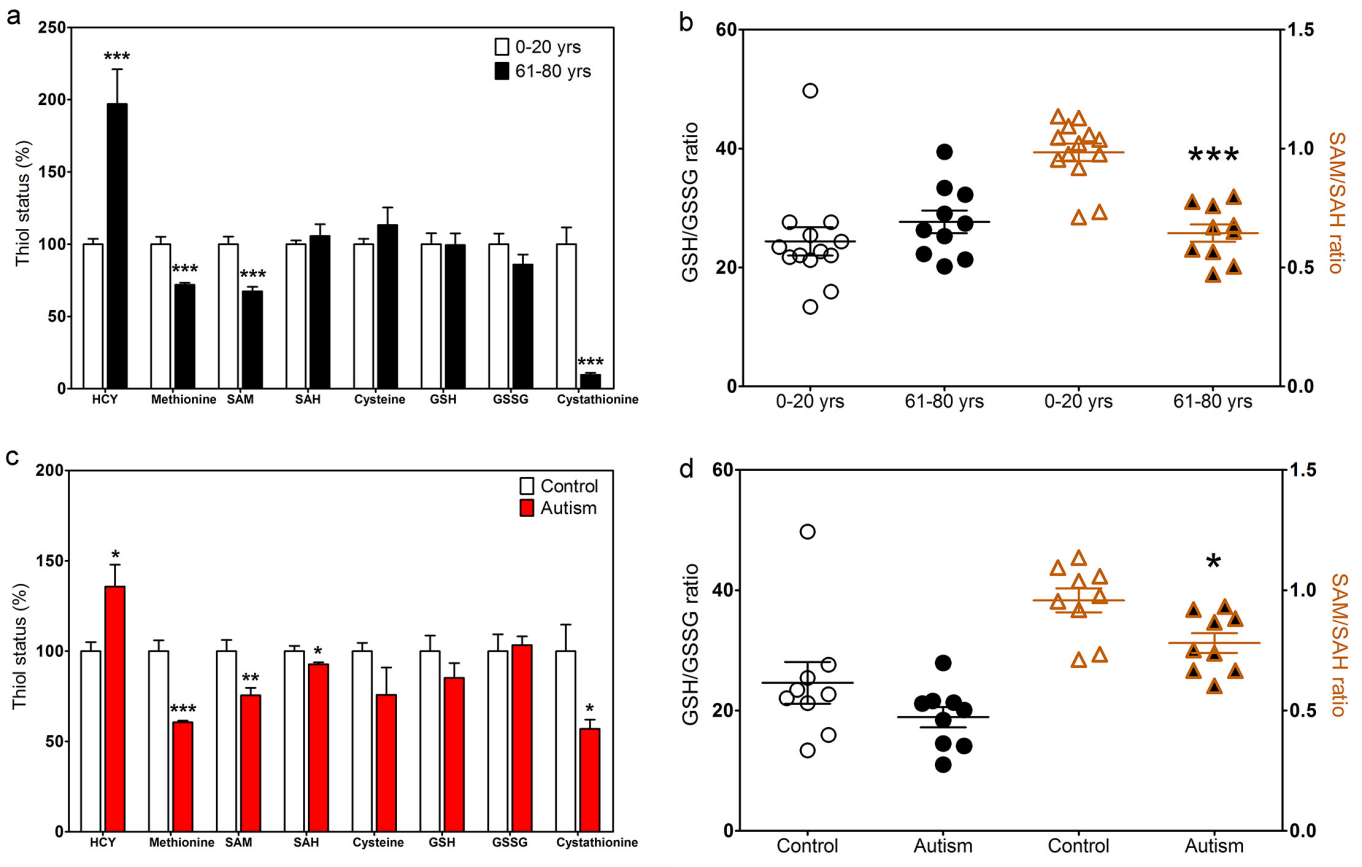
Redox and methylation metabolites in aging and autism. (a) Redox and methylation pathway metabolites in control subjects of 0–20 yrs (n = 12) compared to subjects of 61–80 yrs (n = 10). (b) GSH/GSSG ratio (left) and SAM/SAH ratio (right) in aging. (c) Redox and methylation pathway metabolites in frontal cortex of autistic subjects (n = 9) compared to age-matched controls (n = 9). (d) GSH/GSSG ratio (left) and SAM/SAH ratio (right) in autism. * Indicates a significant difference from 0–20 yrs group (panels a and b) or control group (panels c and d) (* p < 0.05, ** p < 0.01, *** p < 0.001).

Through its role as cofactor for MS, the status of MeCbl can influence the level of methionine methylation cycle metabolites as well as metabolites in the intersecting pathways which provide for GSH synthesis, as illustrated in Fig 1. A comparison of frontal cortex metabolite levels in younger (0–20 yrs) *vs*. older (61 to 80 yrs) subjects revealed several significant differences (Fig 4A). The level of HCY was 2-fold higher in older subjects, while the level of methionine was lower, indicative of lower MS activity. The level of SAM, whose formation is MS-dependent, was also lower in older subjects, in association with a decrease in the SAM to SAH ratio (Fig 4B), indicating an impaired methylation potential. Cysteine, GSH and GSSG levels, as well as the GSH to GSSG ratio, were unaffected by age. Remarkably, the level of cystathionine was 10-fold lower in older subjects. Cystathionine is an intermediate in the transsulfuration of HCY to cysteine and its level is higher in human brain compared to other species, reflecting restricted transsulfuration activity [39,40]. Thus the age-dependent decrease in frontal cortex MeCbl appears to be associated with a loss of this restriction, allowing increased HCY diversion toward GSH synthesis.

A comparison of methionine cycle metabolites in autistic *vs*. age-matched young control subjects revealed a pattern generally similar to older subjects (Fig 4C). Thus HCY was higher, methionine and SAM levels were lower, and GSH and oxidized glutathione (GSSG) levels, as well as the GSH to GSSG ratio, were not different from age-matched controls, while the level of cystathionine was 3.5-fold lower in autistic subjects (Fig 4D). Similar to the influence of aging, the SAM to SAH ratio was significantly lower in autism, indicative of impaired methylation potential in association with an increase in transsulfuration and GSH synthesis at a younger than normal age.

**Fig 5:**
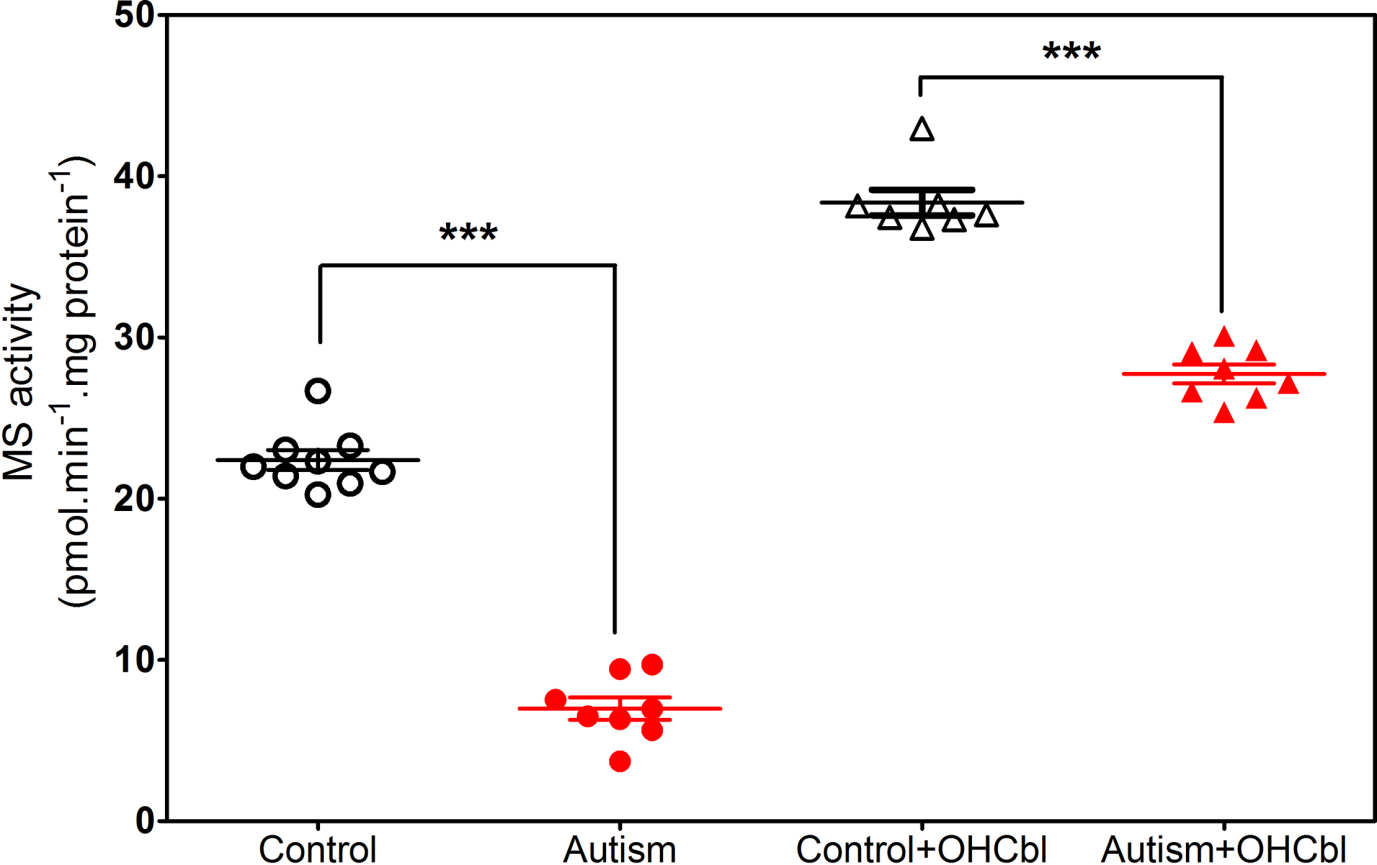
Methionine synthase activity in autism. Methionine synthase activity in frontal cortex of autistic and age-matched control subjects measured either with only endogenous Cbl or with the addition of OHCbl. * Indicates a significant difference from control group (* p < 0.05, ** p < 0.01, *** p < 0.001).

To assess methylation capacity in the context of a deficit in Cbl, we measured MS activity (i.e. conversion of HCY to methionine) in frontal cortex samples of control and autistic subjects with or without provision of exogenous OHCbl. As illustrated in Fig 5, MS activity was 3-fold lower in autistic *vs*. control subjects when only endogenous Cbl was available, and was 38% lower under OHCbl-supplemented conditions, confirming the functional importance of decreased frontal cortex Cbl levels in autism.

### Decreased GSH synthesis lowers brain B12 levels

**Fig 6:**
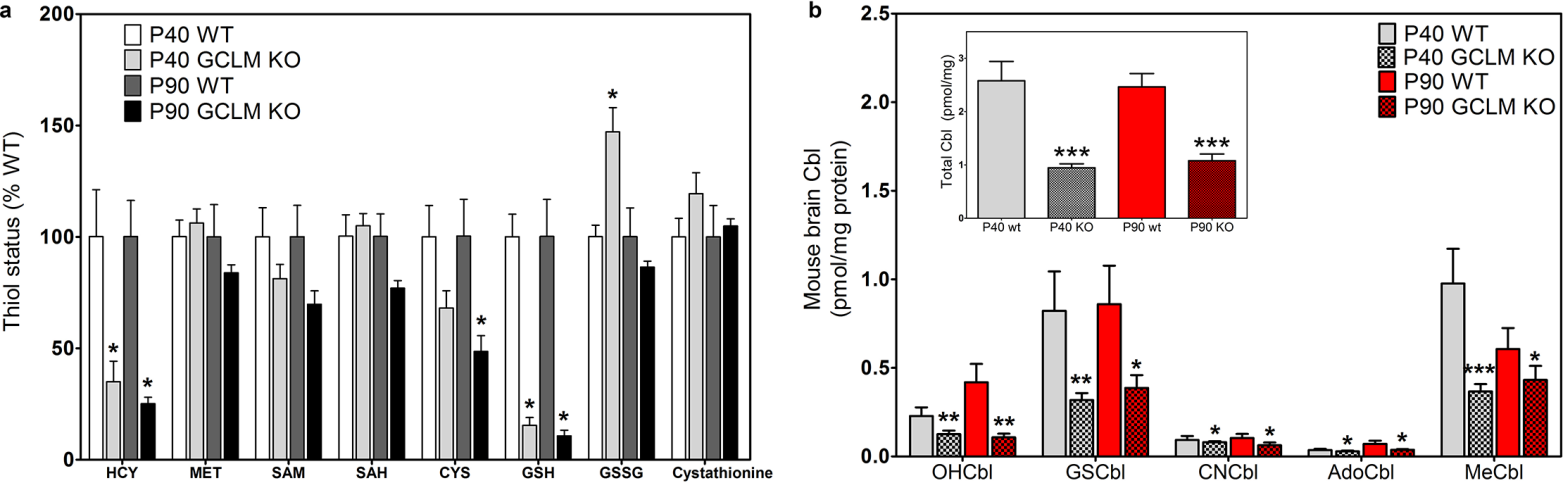
Redox and methylation metabolite and cobalamin status in GCLM KO mice. (a) Redox and methylation metabolite levels in frontal cortex of GCLM KO mice at P40 and P90 (n = 7). Results are expressed as a percentage of the WT level of each metabolite. (b) Levels of five individual Cbl species in frontal cortex of GLCM KO and WT mice at P40 and P90. Inset indicates total Cbl levels. * Indicates a significant difference from control group (* p < 0.05, ** p < 0.01, *** p < 0.001).

Since previous studies have reported lower levels of GSH in autism [27,28] and schizophrenia [29,30], we investigated whether a decrease in GSH affects brain Cbl levels. GCL is the rate-limiting step in GSH synthesis and its modulatory subunit increases GCL activity [41]. Accordingly, we examined thiol metabolite and Cbl levels in cortex of GCLM-KO mice at 40 and 90 days of age, as compared to C57Bl/6J wild-type mice. Consistent with prior studies [42], the level of GSH in frontal cortex of GCLM-KO mice was decreased by 85% and 89% at 40 and 90 days, respectively, in comparison to wild-type mice, along with significant (p < 0.05) decreases in HCY and cysteine (at 90 days), while GSSG was increased at 40 days (Fig 6A). Lower levels of GSH were associated with a significantly lower level of total Cbl (p < 0.001) in GCLM-KO cortex, amounting to 63% and 56% at 40 and 90 days, respectively (Fig 6B inset). The decrease affected all Cbl species, including decreases of 68% and 39% in MeCbl (Fig 6B). An age-dependent decline in the level of MeCbl was evident in cortex of control mice, amounting to a decrease of 38% between 40 and 90 days. Notably, the total Cbl level was approximately 3-fold higher in human (20–40 yrs) *vs*. murine frontal cortex, while MeCbl levels were more than 4-fold higher (*cf*. Figs 2C and 6B).

## Discussion

Here we report novel findings suggesting that levels of vitamin B_12_, especially its MeCbl form, decrease with age in frontal cortex of control human subjects. Since serum Cbl levels do not show a similar decrease with age, our results further suggest that vitamin B_12_ status in the brain compartment is distinctly regulated from the rest of the body and dynamic changes in brain MeCbl across the lifespan may play an important functional role in methylation-dependent processes, including epigenetic regulation of gene expression. Additionally, we observed abnormally lower total Cbl and MeCbl levels in subjects with autism and schizophrenia, as compared to age-matched controls. To our knowledge, this is the first report of pathologically reduced levels of active Cbl species in autistic and schizophrenic brain. Although the number of brain samples analyzed was limited, our findings highlight a possible role for vitamin B_12_-dependent methylation reactions in brain function and in the etiology of neurological disorders.

As a compartment distinct from the rest of the body, the metabolic environment of the brain depends upon the bi-directional transport of nutrients and micronutrients across the choroid plexus neuroepithelial barrier into the CSF. The aging-related decrease in total brain levels of vitamin B_12_ observed in our studies is likely to reflect changes in the activity of one or more of these transport processes across the lifespan, particularly since serum levels do not show a similar decrease^38^. Although the precise molecular basis of vitamin B_12_ transport into CFS is not fully understood, one or more active and selective transport systems are likely to be involved. Transport across the choroid plexus is an important determinant of CSF composition, and megalin, protein product of the *LRP2* gene, which has been implicated in vitamin B_12_ transport by the distal ileum and the renal proximal tubule, is expressed in choroid plexus epithelial cells, along with amnionless and cubilin [14,43]. Binding of Cbl-loaded TC to megalin leading to Cbl uptake has been described [44]. Megalin also facilitates endocytosis of the TCR [45] and TCR knock out mice show a deficit in brain B12 concentrations accompanied by DNA hypomethylation [46]. A recent study showed that *LRP2* is robustly expressed in the distal ileum during fetal and early postnatal development, but is not expressed in adult human ileum [47], indicating an age-dependent decline in its expression. Thus a decline in megalin and/or TCR activity could underlie the lower brain levels of vitamin B_12_ we observed in elderly subjects. Interestingly, megalin promotes removal of amyloid precursor protein-derived Aβ peptide by the choroid plexus and an age-related decline in megalin function has been proposed as a contributor to increased brain levels of Aβ in Alzheimer’s disease (AD) [48]. Impaired MS activity and elevated HCY have been linked to AD and supplementation with vitamin B_12_, folate and vitamin B_6_ decreases progression of cognitive impairment [49–51]. In combination with our results, these observations suggest a coordinated normal decrease in choroid plexus-mediated transport of vitamin B_12_ into the brain and Aβ out of the brain with advancing age (>40 yrs), while environmental and genetic factors introduce increased risk of neurodegenerative disorders in vulnerable individuals.

We found CNCbl to be 15-fold higher in fetal samples, as compared to 0–20 yr old subjects, suggesting unique Cbl metabolism during fetal development. However, the underlying cause of this higher CNCbl level remains unclear, as does the biological origin CNCbl. Maternal folate and vitamin B_12_ supplementation is a common recommendation during pregnancy, which could be a source of the elevated CNCbl we observed, although Cbl levels in placenta were comparatively low (Fig 1F). Conversion of CNCbl to active cofactors MeCbl and AdoCbl requires NADPH- or GSH-dependent decyanation by MMACHC [52,53] and it is possible that the developing fetal brain has diminished decyanation capacity. The markedly higher level of inactive CNCbl could potentially have functional consequences by competing with MeCbl and AdoCbl, restricting their cofactor activity.

MeCbl is the most abundant Cbl species in the brain of younger subjects and its 12-fold decrease was the largest contributor to the 2.7-fold decrease in total Cbl between 0–20 and 61–80 yr old subjects (Fig 2C and 2D). As an essential cofactor for MS, MeCbl availability regulates methylation capacity, reflected as a decrease in the SAM to SAH ratio in elderly subjects (Fig 4B). Among hundreds of methylation reactions which are dependent upon [SAM]/[SAH], methylation of DNA and histones merit special attention for their contribution to epigenetic regulation of gene expression. Dynamic changes in DNA methylation status at specific loci in frontal cortex are closely correlated with chronological age [54], consistent with the age-dependent decrease in MeCbl we observed in frontal cortex. Moreover, changes in DNA methylation provide an epigenetic mechanism of memory formation [55] and the capacity of the brain for learning is therefore a reflection of its ability to modify patterns of DNA and histone methylation and to sustain these patterns over time. D4 dopamine receptor-mediated phospholipid methylation is completely dependent upon MS activity [4] and D4 receptor activation promotes gamma frequency synchronization of neural networks during attention [5,6]. Thus MeCbl-dependent MS activity is poised to play a critical role in both attention and learning.

Autism is a complex neurodevelopmental disorder and a number of studies have reported low plasma levels of GSH and a decrease in [SAM]/[SAH] [56–61], leading us to propose a “Redox/Methylation Hypothesis of Autism” whereby the sensitivity of MS to oxidative stress could lead to impairments in epigenetic regulation and D4 receptor-mediated attention [62]. Our current finding that frontal cortex levels of MeCbl are 3.5-fold lower in autistic subjects *vs*. age-matched controls (Fig 2B) lends support to this hypothesis, linking decreased methylation capacity in the brain to the deficits in neurodevelopment and learning capacity which are hallmarks of autism. Serum and plasma levels of vitamin B_12_ are reported to be normal in autism [60,63], except under conditions of overt nutritional deficiency [64], suggesting that the lower brain levels we observed might result from a limitation in its transport into the brain compartment. While lower serum B_12_ levels have been reported for schizophrenia in several studies [65–67], others found no difference [68,69] or higher levels [70]. In an earlier study we showed that MS mRNA levels in frontal cortex decreased dramatically across the lifespan and levels in autistic subjects were approximately one-half of age-matched controls, although protein levels were not decreased in autistic and elderly subjects [26]. We found that MS enzyme activity is significantly reduced in autistic subjects when measured with endogenous Cbl, and this deficit can be largely, but not completely, reversed by addition of OHCbl (Fig 5). Thus deficits in MS transcription and availability of its vitamin B_12_ cofactor may both contribute to impaired methylation in autism. It remains unclear whether these deficits occur prenatally or postnatally, or if they reflect an acceleration of the normal age-dependent decline caused by one or more environmental factors.

Decreased GSH levels may contribute to impaired vitamin B_12_ transport into the brain, as indicated by lower total Cbl and MeCbl in GCLM-KO mice (Fig 6B). However, while total Cbl and MeCbl levels were lower in frontal cortex of autistic subjects (Fig 3A and 3B), GSH levels were not decreased (Fig 4C). GSH levels have been reported to be decreased in some brain regions (e.g. cerebellum and temporal cortex) in autism, but not decreased in other regions (e.g. frontal, parietal and occipital cortex) [29,30]. The metabolic basis for these regional differences remains obscure, but may relate to their different functional roles. For example, some brain regions may be metabolically keyed to maintain GSH/GSSG (stable redox status), while other regions may maintain SAM/SAH (stable methylation status). We speculate that the former would exhibit more dynamic methylation-dependent epigenetic responses and a higher level of neuroplasticity, while epigenetic stability in the latter would favor memory. We recently showed that vitamin B_12_ levels in cultured human neuronal cells is strongly linked to GSH levels and neurotrophic factor activation of the PI3 kinase signaling pathway augments GSH synthesis in parallel with increased MeCbl and AdoCbl levels [71]. Taken together, our results suggest that the well-documented systemic deficit of GSH in autism, as measured in the blood [56–61], may be linked to decreased vitamin B_12_ transport into the brain. However, the GCLM-KO model, which genetically restricts GSH synthesis throughout the body, does not replicate our brain findings in autistic subjects. Further studies are needed to assess the impact of systemic GSH depletion on vitamin B_12_ transport into the brain.

A GSH deficit has been proposed to be a key factor in the etiology of schizophrenia [30–32] and GCLM-KO mice have been extensively characterized as an animal model showing many schizophrenia-related phenotypes [42,72–74]. GCLM-KO mice exhibit a significant decrease in γ-frequency synchronized oscillations, a shared feature of schizophrenia and autism [75], and D4 dopamine receptor activation in parvalbumin-expressing GABAergic interneurons is essential for synchronized γ oscillations [76]. Since D4 receptor-mediated phospholipid methylation is absolutely dependent upon MS activity [4], lower levels of MeCbl may contribute to diminished γ-frequency synchronized oscillations in autism and schizophrenia. Indeed, autism was initially described as “childhood onset schizophrenia” and these two disorders share many risk genes and core psychiatric/neurological features [77].

The finding of decreased brain vitamin B_12_ in autism is analogous to cerebral folate deficiency (CFD) syndrome [78], and approximately 75% of autistic subjects exhibit autoantibodies capable of blocking folate receptor-mediated folate transport in the choroid plexus [79]. Megalin-directed autoantibodies are relatively common in autoimmune diseases [80], decreasing its function could restrict transport of both folate and vitamin B_12_, combining to limit MS and methylation activity in the brain. Decreased levels of total vitamin B_12_ and MeCbl in autistic subjects (average age 7.5 yrs), were accompanied by a pattern of methylation and transsulfuration metabolites more typical of control subjects 50–60 yrs of age (*cf*. Figs 1C and 2B), similar to the premature decrease in MS expression we previously reported (26), which can be expected to have neurodevelopmental consequences.

Low levels of MeCbl may help explain long-standing observations of abnormal single-carbon metabolism in schizophrenia. For example, as reviewed by Cohen *et al*. [81], more than ten studies demonstrated that intake of 20 mg of L-methionine/day induces an acute psychotic reaction in 40% of schizophrenic individuals, but is without effect in normal subjects_._ We previously reported that phospholipid methylation is significantly lower in lymphocytes from schizophrenic subjects [4] and similarly abnormal epigenetic patterns are present in both lymphocytes and corticolimbic brain regions [82]. Moreover, the age-dependent decline in MeCbl we observed in this study, in conjunction with the previously reported decline in MS transcription [26] may be responsible for the characteristic post-adolescence onset of schizophrenia, which is associated with elevated levels of HCY [83].

Individual risk for brain disorders associated with the age-dependent decrease in MeCbl may depend upon genetic factors affecting methylation capacity. In accord with this notion, single-nucleotide polymorphisms (SNPs) in genes for methionine synthase (*MTR*), methionine synthase reductase (*MTRR*), transcobalamin (*TCN2*) and 5,10-methylenetetrahydrofolate reductase (*MTHFR*), which limit their respective activities, are associated with increased risk of autism [57,84] and schizophrenia [83,85,86], as well as major depression and bipolar disorder [85,86], Parkinson’s disease [87] and Alzheimer’s disease [86,88]. The breadth of these disorders indicates a central role for methylation in maintaining normal brain function and suggests that vulnerability to brain disorders at different stages of life involves impaired methylation, in combination with other risk factors specific to a particular disorder. Indeed, restriction of methylation-dependent epigenetic regulation may enhance the risk of genetic variants which might otherwise be benign.

While provision of supplemental vitamin B_12_ may be helpful in treating the aforementioned brain disorders, several issues must be considered. The required dosage may significantly exceed the Recommended Dietary Allowance (RDA) of 2.4 μg/day. Adequate absorption from the GI tract is essential for oral dosage and transport across the choroid plexus is critical for raising brain levels. Nasal administration may provide an alternative route of administration. The supplemented form of vitamin B_12_ (commonly CNCbl) must be converted to the active MeCbl and AdoCbl species, which requires adequate levels of GSH and NADPH, and a functional vitamin B_12_ deficiency state may result when levels of these reducing factors are low, as in oxidative stress [89]. Thus supplementation with supraphysiological levels of the active Cbl species (i.e. MeCbl and AdoCbl) may be required to address an oxidative stress-related functional deficiency [90].

Our findings are subject to several limitations, most important of which is the number of samples analyzed. A larger study is warranted, particularly with regard to autism and schizophrenia samples, although availability of the former is particularly limited. Despite this limitation, the differences we observed are robust. We were not able to measure serum and brain B_12_ levels of the same subjects, which would provide more definitive comparison of age-dependent changes. Such studies could be carried out in animals. We did not investigate gender differences, which would be particularly relevant for autism, since it is more prevalent in males. The changes we observed in frontal cortex may not occur in other brain regions, so our findings should not be generalized to the entire brain, subject to further studies. The absence of further demographic or nutritional data, including the use of vitamin supplements, did not allow us to evaluate their potential contribution to brain B_12_ levels.

In conclusion, vitamin B_12_ levels in human frontal cortex decrease with age, especially MeCbl, which plays a crucial role in regulating all methylation reactions, including those providing epigenetic regulation of gene expression. MeCbl deficits in autistic and schizophrenic subjects suggest that impaired methylation may be a critical pathological component of these brain disorders, as well as other neurological and neuropsychiatric conditions. Our findings provide a novel redox/methylation-based perspective on the metabolic systems which support normal brain function across the lifespan.

## Supporting Information

S1 File Clinical details for control, autism and schizophrenia subjects. (DOCX)

S2 File Representative chromatograms for cobalamin standards and brain samples. (DOCX)
