# Supplementary material for: Decreased Brain Levels of Vitamin B12 in Aging, Autism and Schizophrenia
Source: PLoS One. 2016 Jan 22;11(1):e0146797. doi: 10.1371/journal.pone.0146797 (PMC4723262; doi:10.1371/journal.pone.0146797)
Supplement: S1 File — (DOCX) [file pone.0146797.s001.docx]

**S2 File: Tissue sample clinical details.** Control subject clinical details (**Table A**). Control and autistic subject clinical details (**Table B**). Control and schizophrenic subject clinical details (**Table C)**.

| **Table A Control subject clinical details.** | | | | |
| --- | --- | --- | --- | --- |
| **Case no.** | **Sex** | **Age (yrs)** | **PMI (hrs)** | **Cause of death** |
| 250 | M | 19 (wks) | 1 | Unknown |
| 4715 | M | 20 (wks) | 4 | Unknown |
| 4654 | Unknown | 20 (wks) | 2 | Unknown |
| 637 | F | 39 (wks) | 2 | Hypoplastic left heart |
| 4670 | M | 5 | 17 | Commotio cordis |
| 4332 | M | 5 | 18 | Pseudomonas brochopneumonia |
| M3415M | M | 8 | 12 | Multisystem failure |
| 4337 | M | 8 | 16 | Blunt force neck injury |
| 1708 | F | 8 | 20 | Compressional asphyxia and multiple injuries |
| 5391 | M | 9 | 12 | Drowning |
| 5161 | F | 11 | 22 | Accident, hanging |
| 5334 | M | 13 | 15 | Hanging, suicide |
| 5387 | M | 13 | 13 | Drowning |
| 5446 | F | 17 | 18 | Acute reactive airway disease |
| 6396 | M | 18 | Unknown | Unknown |
| 914 | M | 20 | 18 | Accident, multiple Injuries |
| 5342 | M | 23 | 14 | Multiple injuries |
| 5346 | F | 25 | 14 | Multiple blunt force injuries |
| 4345 | F | 28 | 19 | Respiratory failure |
| 6162 | M | 36 | Unknown | Unknown |
| 6076 | M | 38 | Unknown | Unknown |
| 6077 | M | 43 | Unknown | Unknown |
| 4103 | M | 43 | 23 | Atherosclerotic Hearth Disease |
| 3748 | M | 44 | 23 | Rectal bleeding |
| 6104 | M | 46 | Unknown | Unknown |
| 4192 | M | 46 | 25.9 | Unknown |
| 3864 | M | 47 | 18.8 | Accident |
| 4739 | M | 50 | 26.5 | Septicemia |
| 5399 | M | 51 | 17 | Dilated cardiomegaly |
| 1568 | F | 51 | 22 | Pulmonary embolism |
| 5456 | M | 58 | 16 | Atherosclerotic cardiovascular disease |
| 5080 | F | 61 | 14 | Multiple injuries |
| 5452 | M | 68 | 23 | Occlusive pulmonary thromboembolism |
| 5082 | M | 68 | 19 | Atherosclerotic cardiovascular disease |
| 4789 | F | 72 | 19 | Accident, exsanguination |
| 4735 | M | 74 | 21 | Chronic obstructive pulmonary disease |
| 4921 | F | 74 | 13 | Peritonitis |
| 1818 | M | 77 | 3 | Atherosclerotic cardiovascular disease |
| 5219 | F | 77 | 3 | Cancer |
| 5246 | F | 78 | 25 | Atherosclerotic cardiovascular disease |
| 1569 | F | 77 | 8 | Drowning complicating HASCVD |
| 5171 | M | 79 | 5 | Chronic obstructive pulmonary disease |
| 5444 | M | 80 | 10 | Hypertensive Cardiovascular Disease |

| **Table C Control and schizophrenic subject clinical details**. | | | | | |
| --- | --- | --- | --- | --- | --- |
| **Case no.** | **Sex** | **Age (yrs)** | **PMI (hrs)** | **Brodmann Area** | **Cause of death** |
| **Controls:** |  |  |  |  |  |
| 6162 | M | 36 | Unknown | 10 | Unknown |
| 6076 | M | 38 | Unknown | 10 | Unknown |
| 6077 | M | 43 | Unknown | 10 | Unknown |
| 4103 | M | 43 | 23 | 9 | Atherosclerotic Hearth Disease |
| 3748 | M | 44 | 23 | 9 | Rectal bleeding |
| 6104 | M | 46 | Unknown | 10 | Unknown |
| 4192 | M | 46 | 25.9 | 10 | Unknown |
| 3864 | M | 47 | 18.8 | 10 | Accident |
| 4739 | M | 50 | 26.5 | 10 | Septicemia |
| **Schizophrenia:** |  |  |  |  |  |
| 5787 | M | 36 | Unknown | 10 | Unknown |
| 3564 | M | 42 | 14.2 | 10/9 | Cancer |
| 3259 | M | 44 | 17.8 | 10 | Suicide |
| 5600 | M | 46 | Unknown | 10 | Unknown |
| 6545 | M | 46 | Unknown | 10 | Unknown |
| 5293 | M | 46 | 29.1 | 10 | Cancer and bowel peritonitis |
| 3597 | M | 46 | 29.5 | 10 | Cancer |
| 5327 | M | 47 | Unknown | 10 | Unknown |
| 5115 | M | 49 | 24.5 | 10 | Acute respiratory disease |
