# Supplementary material for: Decreased Brain Levels of Vitamin B12 in Aging, Autism and Schizophrenia
Source: PLoS One. 2016 Jan 22;11(1):e0146797. doi: 10.1371/journal.pone.0146797 (PMC4723262; doi:10.1371/journal.pone.0146797)
Supplement: S2 File — (DOCX) [file pone.0146797.s002.docx]

**S2 File: Representative chromatograms for cobalamin standards and brain samples.** HPLC separation of six cobalamin standards (injection volume 30 µl of 10 µM solution). While sulfitocobalamin (SO_3_Cbl) could be detected, it was not reported since its level was very low (~3 % of total) in all samples and no significant group to group differences were observed **(Figure A)**. Electrochemical detection-based chromatogram for a cobalamin-containing extract from frontal cortex of an 8 year-old male control subject (**Figure B**). Electrochemical detection-based chromatogram for a cobalamin-containing extract from frontal cortex of a 77 year-old male control subject (**Figure C**). Electrochemical detection-based chromatogram for a cobalamin-containing extract from frontal cortex of an 8 year-old male autistic subject (**Figure D**). Electrochemical detection-based chromatogram for a cobalamin-containing extract from frontal cortex of a 46 year-old male schizophrenic subject (**Figure E**).

**Figure A**

**Figure B**

**Figure C**

**Figure D**

**Figure E**
